# The Use of a Biologically Transparent Illumination Guide to Detect Gastric Tube Tip Position in Young Children From Diverse Clinical Backgrounds

**DOI:** 10.7759/cureus.76871

**Published:** 2025-01-03

**Authors:** Takato Sasaki, Yuri Nemoto, Aya Nishigata, Tomohiro Aoyama, Kouji Masumoto

**Affiliations:** 1 Department of Pediatric Surgery, Institute of Medicine, University of Tsukuba, Tsukuba, JPN

**Keywords:** biologically transparent illumination system, biologically transparent light, gastric tube, gastric tube tip verification, pediatrics

## Abstract

To confirm that the tip of a gastric tube is properly positioned within the stomach, commonly used methods include simple X-ray examination, the auscultation of gastric bubble sounds, and pH testing of aspirated fluids. However, these methods have limitations, such as concerns regarding radiation exposure and accuracy. We utilized the Tumguide^® ^(Otsuka Pharmaceutical Factory, Inc., Tokushima, Japan), a gastric tube tip verification system that employs biologically transparent (BT) red light-emitting diode (LED) light, for gastric tube insertion in five pediatric patients with conditions considered unfavorable for BT light transmission. Based on our experience, we report key considerations regarding its indications and applications. In all cases, the system was used under general anesthesia during preoperative gastric tube insertion for the patients' underlying conditions. Gastric tube insertion was successfully performed without complications in all cases. However, in one case involving the use of an 8 Fr Salem Sump™ tube (Cardinal Health K.K., Tokyo, Japan) for decompression, a difficulty arose in removing the fiber. This issue was resolved by removing the fiber along with the tube, filling the tube with water, and reinserting it. In a girl with jaundice due to biliary atresia (serum total bilirubin level of 8.7 mg/dL) and a boy with dark skin, the visibility of the BT light was reduced due to skin pigmentation. However, dimming the room lights enhanced visibility. In one case where a colostomy overlapped the stomach and in two cases where preoperative CT scans confirmed that the left lobe of the liver overlapped the stomach, the BT light was clearly visible, enabling accurate guidance and the confirmation of the tube tip position. Postoperative X-ray examinations were conducted in four out of the five cases, confirming the correct placement of the tube tip in the stomach.

Based on our experience and previous reports, the BT light-based gastric tube tip verification system, which uses red LED light to confirm the position of the nasogastric tube tip, has proven to be an effective tool for the real-time verification of nasogastric tube placement in young children.

## Introduction

In pediatric care, as in adult care, nasogastric or orogastric tubes (hereafter referred to as "gastric tubes") are commonly used for gastric decompression or enteral nutrition. However, the insertion of gastric tubes carries significant risks, particularly when the tube is mistakenly placed in the airway. Such misplacement can lead to severe complications, including aspiration pneumonia and pneumothorax [1]. The risk of misplacement is reported to be particularly high in young children [2]. Therefore, confirming the correct placement of the gastric tube tip is essential. Commonly used verification methods include X-ray imaging, ultrasonography, auscultation, pH testing of aspirates, and capnography [3]. However, these methods have notable limitations. For example, X-ray imaging poses a risk of radiation exposure, while the reliability of other methods has been questioned.

The biologically transparent (BT) light-based gastric tube tip verification system addresses these challenges by utilizing highly bio-permeable red light. This system detects BT light emitted from a fiber located at the tip of the gastric tube from outside of the body, enabling the confirmation of the tube tip's appropriate placement in the stomach.

In this study, we evaluated the utility and practical considerations of the BT light-based gastric tube tip verification system in five young pediatric patients with diverse clinical conditions.

## Case presentation

Materials and methods

In this analysis, the Tumguide®, a BT light-based gastric tube tip verification system manufactured by Otsuka Pharmaceutical Factory, Inc. (Tokushima, Japan), was used to confirm the position of the gastric tube tip. This device employs BT light (wavelength: 660 nm), which has high tissue penetration. BT light passes through soft tissues while being blocked by hard tissues such as bone and cartilage [4]. Leveraging this property, BT light has been used in other applications, such as determining the position for percutaneous sigmoid colon fixation to assist with endoscopy [5]. Reports on the use of BT light-based gastric tube tip verification system in pediatric cases are extremely limited [6,7]. To ensure the safe placement of the tube in infants and young children, all procedures were performed under general anesthesia during gastric tube insertion, as part of intraoperative and postoperative management associated with each patient's underlying condition.

**Figure 1:**
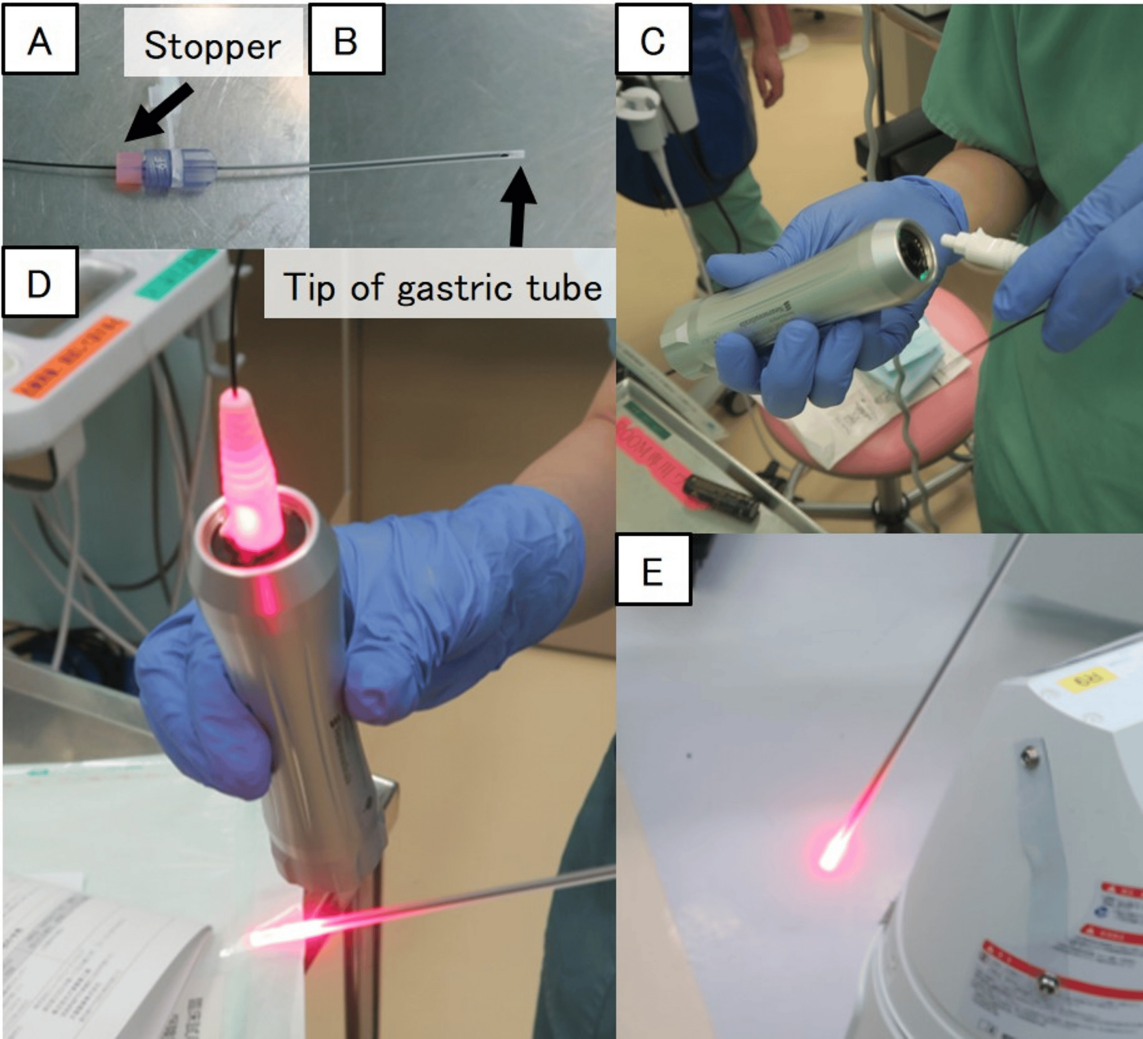
Preparation of the BT light-based gastric tube tip verification system (Tumguide®) before tube insertion into the stomach (A and B) Carefully insert the fiber into the gastric tube, ensuring that its tip remains fully contained within the tube. Once properly positioned, secure the fiber at the base using a stopper. (C-E) Connect the fiber to the light source device, and activate the power. Ensure that the red light is clearly emitted from the fiber at the tip of the gastric tube before proceeding with its use BT: biologically transparent

The Tumguide® system consists of a light source device and a disposable, sterile fiber. During the use, the fiber is inserted into the gastric tube without extending beyond its tip, ensuring that the tip remains positioned at the distal end of the tube. Typically, the red light transmitted through the body is visible in the neck and abdomen but becomes almost indiscernible in the thoracic region. In our patients, guided by the red light emitted from the tube tip, the nasogastric tube was inserted as shown in Figure 1.

The Tumguide® system was utilized in a total of five cases (three boys and two girls). For cases where preoperative CT scans were performed, anatomical characteristics were assessed using the imaging. In cases requiring postoperative X-ray examinations to check for retained gauze or other foreign objects, the position of the gastric tube tip was also evaluated using X-ray imaging.

Result

Details of the five cases included in the study are presented in Table 1. Gastric tube placement was successfully achieved in all five cases. However, in one case where an 8 Fr Salem Sump™ tube (Cardinal Health K.K., Tokyo, Japan) was used for decompression instead of a 6 Fr feeding catheter, difficulty in removing the fiber occurred. Consequently, the fiber had to be removed together with the tube. Upon reinserting the gastric tube, after filling it with water, the fiber was removed without any difficulties. In the other four cases, a 6 Fr feeding catheter (JMS, Inc., Tokyo, Japan) was used as the gastric tube without any problems.

**Table 1:** Patient characteristics BT: biologically transparent

Case	Age (month)	Sex	Body weight (kg)	BMI	Primary diagnosis	Factors that may inhibit BT light	Catheter used (as a gastric tube)
1	3	Female	5.8	15.9	Biliary atresia	Jaundice (serum total bilirubin level of 8.7 g/dL)	6 Fr feeding catheter
2	10	Male	7	16.2	Anorectal malformation	The stoma was positioned overlapping the stomach	6 Fr feeding catheter
3	28	Female	12	16.2	Neuroblastoma	The left lobe of the liver was positioned overlapping the stomach	6 Fr feeding catheter
4	57	Male	22.8	18.5	Phimosis	Dark skin color (Fitzpatrick skin types IV-V)	8 Fr Salem Sump™ tube
5	61	Male	14.7	15.3	Congenital biliary dilatation	The left lobe of the liver was positioned overlapping the stomach	6 Fr feeding catheter

**Figure 2:**
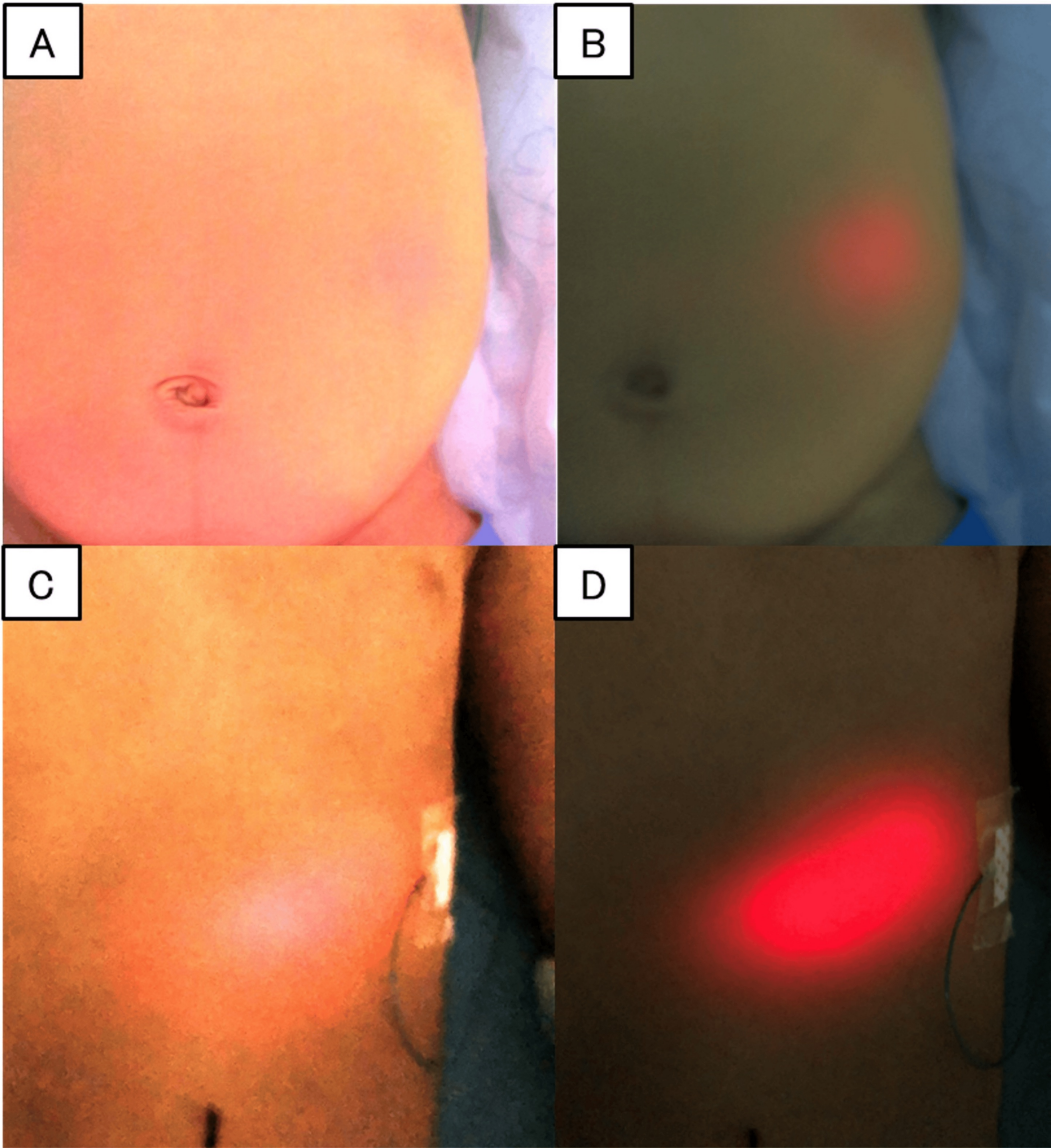
Findings in cases with jaundice or darker skin color (A and B) Case 1 (with jaundice): in this case, jaundice (serum total bilirubin level: 8.7 mg/dL) made it difficult to visualize the BT light emitted from the stomach in a brightly lit environment. However, dimming the surrounding lights improved visibility, enabling the precise identification of the nasogastric tube tip position. (C and D) Case 4 (with dark skin color): similarly, in this case, darker skin pigmentation (Fitzpatrick skin types IV-V) also posed a challenge for visualizing the BT light in a brightly lit environment. By dimming the surrounding lights, the BT light became clearly visible, allowing the accurate identification of the nasogastric tube tip position BT: biologically transparent

**Figure 3:**
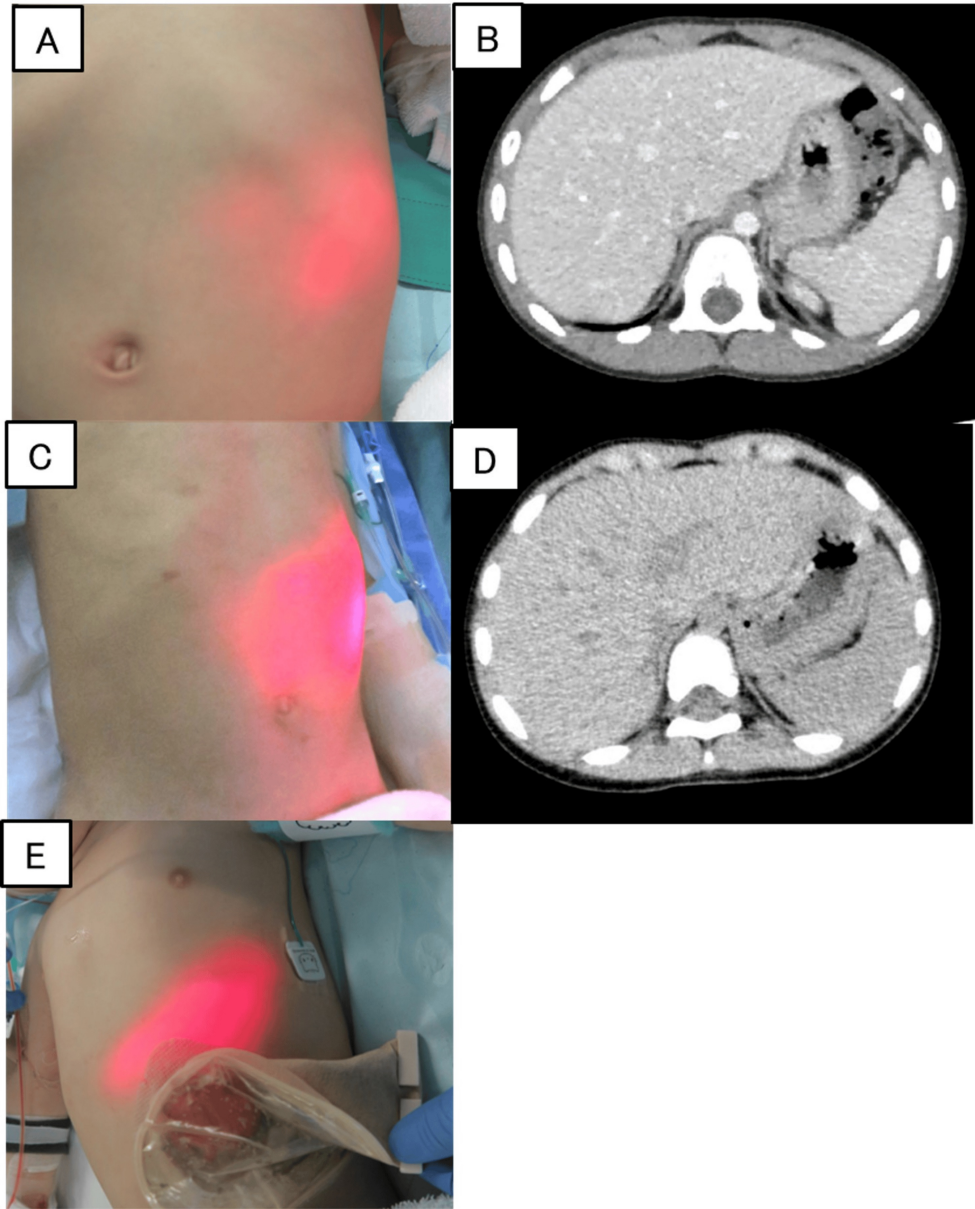
Findings in cases with other organs positioned anterior to the stomach (A-D) In cases 3 (A and B) and 5 (C and D), preoperative CT scans revealed that the left lobe of the liver was positioned anterior to the stomach. Despite this anatomical configuration, the BT light emitted from within the stomach was easily visible on the body surface, without any problems. (E) In case 2, a transverse colostomy was positioned anterior to the stomach. Despite this anatomical arrangement, the BT light emitted from within the stomach was clearly visible on the body surface without any problems BT: biologically transparent

In a girl with jaundice due to biliary atresia (serum total bilirubin level of 8.7 mg/dL) and a boy with darkly pigmented skin, the visibility of the red transillumination light was recognized to be slightly reduced, likely due to skin color (Figure 2). In both cases, enough visualization was achieved by dimming the room lights. In one case where a transverse colostomy overlapped the stomach in the left upper abdomen and in two cases where preoperative CT scans revealed that the left lobe of the liver overlapped the anterior aspect of the stomach, the red transillumination light successfully penetrated the abdominal wall and was clearly visible on the body surface (Figure 3). In all five cases, the appropriate guidance and placement of the gastric tube were successfully achieved.

Postoperative X-ray examinations were performed in four of the five cases, confirming the appropriate intragastric placement of the gastric tube tip in all four cases. In the remaining case, imaging evaluation was not conducted. However, based on the gastric juice excretion through the tube during the operation, the tube placement was considered successful.

## Discussion

In this report, we confirmed the position of gastric tube tips using the BT light system in five cases with differing clinical backgrounds, demonstrating its utility as a gastric tube tip verification system. Traditionally, methods for identifying the gastric tube tip position have included auscultation, the pH testing of aspirated fluid, ultrasonography, and CO_2_ confirmation using a capnometer, as an alternative to X-ray examination (Table 2).

**Table 2:** Advantages and disadvantages, sensitivity, and specificity of methods for gastric tube tip verification Reference list related to each item: ultrasonography [8,9], pH testing of aspirated fluids [9], auscultation [9,10], capnography [11], and BT light system [7,10] PPI, proton pump inhibitor; GER, gastroesophageal reflux; BT, biologically transparent

Method	Advantage	Disadvantage	Sensitivity and specificity
X-ray examination	Accuracy	Radiation exposure, time and cost, and labor demands	Both are basically 100%
Ultrasonography	Easily performed at the bedside	Requires proficiency in evaluation; accuracy	Sensitivity: 86%-100%; specificity: 67%-100% (adults)
pH testing of aspirated fluids	Easily performed at the bedside	False negatives due to antacids/PPI, false positives due to GER, and accuracy	Sensitivity: 86%-100%; specificity: 67%-100% (adults)
Auscultation	Easily performed at the bedside; almost no cost	Accuracy	Sensitivity: 98%-100%; specificity: 33%-67% (adults)
Capnography	Easily performed at the bedside	Accuracy; false negatives caused by tube obstruction	Sensitivity: 80%; specificity: 87% (adults)
BT light system	Easily performed at the bedside; accuracy	Low sensitivity (73%) in obese adults	Sensitivity: 99%; specificity:100% (pediatrics)

However, these methods raise concerns regarding accuracy, making X-ray verification the gold standard [3]. Despite its reliability, X-ray verification presents challenges, including radiation exposure, the time required for testing and confirmation, and associated labor demands. These challenges are particularly significant in pediatric cases, where the effects of radiation are more pronounced than in adults. Therefore, minimizing radiation exposure is emphasized as a critical consideration in pediatric care whenever possible.

In this context, the BT light-based gastric tube tip verification system, as used in this clinical observation, offers a non-radiative, simple, and visual method for confirming the gastric tube tip position within the stomach. Regarding its utility, Satake et al. [7] reported a sensitivity of 99% in a study involving 106 pediatric patients under 16 years of age, which is higher than the sensitivity reported in adults (77.4%-88.8%) [10,12]. The difference in sensitivity is thought to be due to abdominal wall thickness. In this report, we also examined cases with conditions considered unfavorable for BT light transmittance, such as the liver positioned anterior to the stomach, a colostomy overlapping the stomach, jaundice, or darker skin color due to racial characteristics. Our findings indicated that while light visibility was slightly reduced in cases with jaundice or darker skin color, using a darkened environment allowed for enough observation without any problems. No specific issues were encountered when using this system to confirm the gastric tube tip position. To ensure the reliable use of this system, maintaining as dark an environment as possible could facilitate its application across all racial backgrounds, including individuals with darker skin color.

Additionally, this clinical observation utilized two types of gastric tubes: a 6 Fr feeding catheter and an 8 Fr Salem Sump™ tube for decompression purposes. In four cases using the 6 Fr feeding catheter, no problems occurred when removing the fiber optic light. However, in one case using the 8 Fr Salem Sump™ tube, difficulty in removing the fiber was encountered. Previous reports have employed feeding catheters sized 8-12 Fr and Salem Sump™ tubes sized 14-18 Fr, with no reported issues regarding fiber removal [6,7,10,12]. Our observation suggests that compatibility between the catheter's diameter and material may influence fiber removal, highlighting the importance of pre-use preparation, such as lubrication.

The BT light-based gastric tube tip verification method offers notable advantages over X-ray examination, as it eliminates radiation exposure and can be performed conveniently at the bedside. Additionally, it demonstrates superior accuracy compared to other methods, including auscultation, the pH testing of aspirated fluid, ultrasonography, and capnography. However, several disadvantages have been identified, including the cost (¥400,000 {approximately $2,700} for the device and ¥2,000 {approximately $13} per disposable fiber) and reduced sensitivity in obese patients [9]. Nevertheless, Munera-Seeley et al. reported that capnography costs $23 per use, while X-ray examinations cost $795 per session [13]. Considering these comparisons, the running costs of the BT light system seem to fall within an acceptable range.

## Conclusions

This clinical observation, though limited in sample size, provided a detailed evaluation of the BT light-based gastric tube tip verification system in patients with diverse clinical backgrounds. Additionally, a precaution regarding the use of this system in young pediatric cases was identified. While further experience is needed for applications such as in non-anesthetized patients and obese patients, the biologically transparent illumination system is considered a widely applicable and valuable device for pediatric gastric tube tip verification.
